# Carry-over effects of priming viewers with pro and anti-establishment messages in video content

**DOI:** 10.1016/j.heliyon.2024.e27895

**Published:** 2024-03-15

**Authors:** Azi Lev-On, Nili Steinfeld

**Affiliations:** School of Communication, Ariel University, Israel

**Keywords:** Videos, Experiments, Activism, Media effects, Persuasion, Media carryover effect

## Abstract

In many civic domains we witness “video exchanges” between citizens and the establishment; for example, when citizens upload documentation of police violence, and the police uploads documentation from body cameras providing different takes of the incident. Can such videos influence public opinion? We studied if viewing visual content (of a murder reenactment) with pro-prosecution, pro-defense, and no-narration- affects viewers' opinions. We found that not only were viewers' opinions of innocence/guilt and police functioning were affected, but the experience carried over to change opinions about the functioning of the state attorney's office and the courts-*which were not referenced in the videos*. We conclude by discussing the implications for opinion formation in the contemporary media environment.

## 1 Introduction

People's abilities to create and distribute videos to large publics has increased dramatically. Messages of protest or solidarity, pro-establishment and anti-establishment which in the not-too-distant past were produced by large organizations, are increasingly created and distributed by small content producers, reaching far.

Members of oppositional groups have conveyed their messages in many ways, from leaflets to street gatherings. Recently social media has become central arenas for protesters [1,2], where their message take a more visual shape [3,4]. But we know little about the impact of video-activism on opinion change, especially in anti-establishment contexts.

To study this impact, we look at the activity for justice for Roman Zadorov in Israel. Zadorov was convicted of murder and sentenced to life in prison. However, Israelis highly doubt the verdict. Consequently, an intensive social media activity called for justice for Zadorov, including many activist videos Interestingly, video-activism by citizens was followed by video activity by the Ministry of Justice [5].

Such “video exchange” between citizens and establishment is found in many civic domains; for example, when citizens upload documentation of police violence, while the police uploads documentation from policemen body cameras providing a different take of the incident [6]. Are the efforts of activists and the establishment justified? Do these videos impact viewers' opinions?

### 1.1 Why study the visual dimension of activism

The paper studies the impact of exposure to *visual content*, for a few reasons. First, visual information is *processed* in a “faster” path than textual information [7], it is better *retained* [8] and assists to more significant *attitude-changes* (Paivio & Csapo, 1993)*, emotional affect* [9] and *focus* on content and action-than textual information [10].

Second, priming video content can “carry-over” to produce opinion change in related domains. Carryover effects can result from exposure to non-visual content [[11], [12], [13]], but exposure to visual content can arguably produce a more significant carryover effect than exposure to non-visual content (Domke, Perlmutter & Spratt, 2002).

Third, literature in the *legal* domain recognizes the influence of exposure to visual content on conduct of stakeholders in legal proceedings [14]. Research on *social movements* demonstrates its importance for attracting the media's and activists' attention, and for mobilizing them [15].

The focus on video content is also justified by its growing dominance online. For example, YouTube is the most popular social media in the US in 2021 [16], ranking second in global Internet traffic and engagement. Instagram and TikTok are especially popular among young users [17]. Such platforms successfully match video content to users' preference, including niche content that otherwise would go unnoticed [18].

The dominance of the visual is clear in the social media activity for Roman Zadorov-our research environment. Interviews with social media group admins demonstrate that the interest of most was sparked by exposure to visual materials [19]. Visual content was most popularity in these groups [20].

The relevant state institutions also find the visual dimension important. For example, videos from the reenactment of the murder were uploaded to YouTube by the Ministry of Justice [5].

Thus, the public debate regarding Zadorov's guilt has shifted from the textual dimension, the norm in legal domains (requests, summaries, rulings), to the visual.

### 1.2 DIY videos

The increase in people's abilities to upload videos, process them and engage with them is a part of the broader Do-It-Yourself (DIY) trend involving, for example, podcasts, music, or videos self-produced by non-professionals using software, hardware, techniques and knowledge that were previously the domain of skilled experts [21]. Content ranges from “creation from scratch” using materials people have generated and edited, to uploading excerpts from readymade-TV items with minor edits-in order to draw attention to arguments or support narratives [22].

A key element that influences users' involvement in content creation lies in the complexity of the topic being covered. Thus, a series with a complex plot can lead to broad and deep discourse in textual and visual forms [23,24]. The phenomena of DIY, when applied to the legal arena, enables to expand the circle of participants in the processes of production and dissemination of information and knowledge [25].

According to media and law researchers, a structural interdependence exists between journalist who cover the police and the legal domain- and the establishment they cover, that is also their main source of information [26]. Still, in addition to “insiders” such as policemen, lawyers, and reporters-other actors participate in struggles over agenda-setting and framing of legal affairs. The public typically does not have access to legal materials, and its abilities to participate in such struggles, is typically limited. The current study asks what happens when the phenomena of DIY extends to the legal arena.

## 2 Research environment: the social media activism for justice for Roman Zadorov

On December 6, 2006, 13-year-old Tair Rada was found dead at her school in Katzrin, Israel. Roman Zadorov, a flooring installer, was arrested six days later, a week later confessed to it- but shortly recanted, and has since denied any connection to the murder. Zadorov was convicted in 2010 and sentenced to life in prison. The verdict referred to a “high-quality … fabric of evidence” that points to Zadorov, including his confessions to an undercover police informant and to police investigators, and the reenactment of the murder. Zadorov's appeal to the Supreme Court was rejected in 2015.

But opinion polls repeatedly showed overwhelming doubts regarding Zadorov's guilt. A main source of doubts was the victim's mother who repeatedly declared that she doubted whether Zadorov was the killer. Problems in Zadorov's confession and reenactment also contributed to these doubts, as well alternative narratives about the identity of the murderer/s. A key factor influencing the continuing public interest in the affair is the intensive activity for justice for Zadorov on social media. Since 2009 many Facebook groups were established about the case. After Zadorov's appeal to the Supreme Court was rejected (2015), the number of group members soared, and the largest group became one of the largest groups in Israel [27]. The investigation materials were made available on the “Truth Today” website. There are also several dedicated YouTube channels, featuring video materials about the case.

Apart from its scope, the social media activity is unique in other aspects: (1) it is about a murder trial and a call for justice for false convicts-topics that are usually far from the public eye. (2) Typically, participants in the public discourse regarding law and justice are “insiders"- policeman, lawyers, journalists. Here the involvement of “outsiders” is evident. (3) The activity led to many discoveries by activists who pore through the investigation materials, including ones that led to the decision to hold a retrial [5,28].

For these reasons, the activity is a fascinating case for examining the characteristics and effects of social media activism. In 2021 Zadorov was granted a re-trial, and in 2023 he was acquitted.

## 3 Method

### 3.1 Videos

For the experiment we created a 2:30 min long video, based on key parts from the reenactment. The basic video shows Zadorov entering the school, turning to the girls' toilets and demonstrating how he committed the murder.

Participants in the experiment watched one of three versions of the video. The videos were identical except for the narration added to the visual content, and its transcription. The narration excerpts were taken from “Shadow of Truth” true-crime series that presented the defense and prosecution narratives:

1. The pro-prosecution version supported Zadorov's guilt and included narration by Adv. Shila Inbar and Adv. Avi Shai, who was responsible for carrying out the reenactment.
2. The pro-defense version supported Zadorov's innocence and included narration by Advs. David Spiegel and Elkana Leist, Zadorov's defense attornies.
3. no-narration version,

A manipulation check was conducted to determine whether the viewers perceived the narratives emerging from the videos, in-line with researchers' intentions. 131 respondents, who did not participate later in the experiment, were instructed to watch one of the three videos, followed by four questions about Zadorov's guilt, the conduct of the police, the state attorney's office, and the courts (on a 7-point Likert scale). Indeed, there were significant differences between the groups in-line with the experimentally-intended manipulation. Regarding Zadorov's guilt, a post-hoc LSD test demonstrated significant differences between viewers of the pro-defense narration (n = 42, m = 2.26) to viewers of the no-narration video (n = 44, m = 4.05) and viewers of the pro-prosecution video (n = 45, m = 4.71) (F_(2,128)_ = 16.70, p < 0.001).

### 3.2 Procedure

Participants were recruited using a nationwide survey institute (n = 239). We measured participants' opinions in two points. A week prior to watching, participants answered an online questionnaire about their opinions about:

• Their level of agreement with the statement “Roman Zadorov murdered Tair Rada” between “greatly disagree” (1) and “greatly agree” (7). People who chose “no opinion” (0) did not continue to the second stage of the experiment (because we could not measure opinion changes in such cases).
• Three questions regarding the conduct of state institutions: the police/state attorney's office/courts, in this case. Options ranged between “very bad” (1) and “very good” (7).

Respondents also answered questions about their familiarity and interest in the affair. *To allow for measuring possible change in opinions*, we excluded participants who responded (1) or (7) regarding Zadorov's guilt and institutional conduct-from participating in the second stage of the experiment which took place after a week.

In the second phase, participants watched one of the three videos (controlling for age and gender between the groups). Participants then completed a questionnaire identical to the one they filled a week earlier.

## 4 RQs and hypotheses

This study examines if viewing pro- and anti-establishment videos impact viewers' opinion about content *appearing* in the videos-about Zadorov's guilt/innocence and the functioning of the police (i.e. *Direct effects* of watching pro- and anti-establishment videos).

We also examine if viewing pro- and anti-establishment videos impact viewers' opinion about content *not appearing* in the videos-about the functioning of the state attorney's office and the courts (*i.e. Indirect effects* of watching pro- and anti-establishment videos). If such a carryover effect exists then it further validates the claim about the role of activists- and establishment-videos in public opinion formation.

We hypothesize that.

1. Viewers' opinions will change according to the video's inclination. Watching pro-prosecution videos shift viewers' opinion “towards” the prosecution, watching the no-narration video will produce a more moderate opinion change “towards” guilt (because the video shows the murder reenactment which looks “incriminating” without narration suggesting otherwise). Watching the pro-defense video will shift opinions “towards” the defense, strengthening support in Zadorov's innocence.
2. Viewers' opinions regarding the conduct of the institutions involved will also change, *also in reference to the State Attorney's Office and the courts which are not directly addressed in the video.* Watching pro-prosecution videos will produce more *positive* evaluations of the conduct of the police (*direct*), the State Attorney's Office and the courts (*indirect*). Watching the no-narration video will produce more moderate opinion changes in similar directions. Viewing the pro-defense video will produce *negative* changes in evaluating the conduct of the police (*direct*), the State Attorney's Office and the courts (*indirect*).

## 5 Findings

239 people participated in the two rounds of the experiment. 51.5% (123) were males and 48.5% (116) females, ages ranged between 18 and 70 (average = 41.27). Participants reported high levels of familiarity (average = 4.66 of 7) and interest (average = 5.36) in the case. Mean opinion about Zadorov's guilt prior to watching the videos was 3.39 (between 1 and 7 = great agreement with Zadorov's guilt).

Mean opinions about the conduct of state institutions in the case (between 1 = very bad, and 7 = very good) were: 2.72 for the police, 2.94 for state attorney's office, and 3.31 for the courts.

The pre-session variables were compared between the groups, using a series of chi-square and Anova tests. No significant difference between the groups were found. Table 1 presents participants' opinions on Zadorov's guilt and the institutional conduct before and after watching the videos.

**Table 1:** Opinions on Zadorov's guilt and institutional conduct before and after watching the reenactment video.

	Video viewed	Opinions before	Opinions after	t values (paired-sample)
Opinion about Zadorov's guilt (1-Completely Disagree, 7- Completely Agree)				
	Defense-Narration	M = 3.38, SD = 1.94	M = 3.09, SD = 1.9	t_(57)_ = 1.34, p = NS
	No-Narration**	M = 3.19, SD = 1.68	M = 3.70, SD = 1.73	t_(62)_ = -2.85, p < 0.01
	Prosecution-Narration***	M = 3.55, SD = 1.78	M = 4.61, SD = 1.72	t_(61)_ = -5.84, p < 0.001
Opinion about police conduct (1-Very Poor, 7- Very Good)				
	Defense-Narration*	M = 2.78, SD = 1.64	M = 2.43, SD = 1.41	t_(67)_ = 2.2, p < 0.05
	No-Narration**	M = 2.58, SD = 1.39	M = 3.18, SD = 1.72	t_(64)_ = -3, p < 0.01
	Prosecution-Narration***	M = 2.77, SD = 1.43	M = 3.52, SD = 1.62	t_(61)_ = -4.2 p < 0.001
Opinion about state attorney's office conduct (1-Very Poor, 7- Very Good)				
	Defense-Narration	M = 2.89, SD = 1.47	M = 2.78, SD = 1.53	t_(62)_ = 0.85, p = NS
	No-Narration	M = 2.88, SD = 1.49	M = 3.16, SD = 1.59	t_(57)_ = -1.48, p = NS
	Prosecution-Narration**	M = 2.95, SD = 1.25	M = 3.55, SD = 1.54	t_(54)_ = -3.07, p < 0.01
Opinion about courts conduct (1-Very Poor, 7- Very Good)	Defense-Narration	M = 3.17, SD = 1.67	M = 2.95, SD = 1.73	t_(65)_ = 1.54, p = NS
	No-Narration*	M = 3.20, SD = 1.58	M = 3.67, SD = 1.79	t_(59)_ = -2.43, p < 0.05
	Prosecution-Narration***	M = 3.47, SD = 1.64	M = 4.09, SD = 1.8	t_(52)_ = -4.14, p < 0.001

*p < 0.05,**p < 0.01,***p < 0.001.

We found that the inclination of the video significantly affected participant opinions: With regard to Zadorov's guilt (F_(2,180)_ = 12.29, p < 0.001), a post-hoc LSD test shows that the differences are significant between participants who watched the pro-defense video (mean change = −0.29) to those who watched the pro-defense video (m = 0.51) and the pro-prosecution video (m = 1.06). As hypothesized, change was in-line with the video's inclination (i.e. pro-defense or pro-persecution). Additionally, a larger opinion change is evident among viewers of the pro-prosecution video than viewers of the no-narration video.

The same trend repeats in the evaluation of state institutions: Significant differences were found regarding change in evaluation of police conduct (F_(2,192)_ = 11.23, p < 0.001). Mean difference decreased only for viewers of the pro-defense video (m = −0.35), and were significantly lower than difference in the no-narration group (m = 0.6), and in the pro-persecution group (m = 0.74).

The trends repeats regarding change in court evaluation (F_(2,176)_ = 7.77, p < 0.01), with significant differences found between the pro-defense group (m = −0.21), the no-narration group (m = 0.47) and the pro-prosecution group (m = 0.62).

Evaluations of state attorney's office evaluation also differed significantly between the groups (F_(2,173)_ = 4.39, p < 0.05). Here the significant difference is between the pro-defense group (m = −0.11) and the pro-prosecution group (m = 0.6). Opinion change in the no-narration group was insignificant but in the expected direction (m = 0.28).

The findings demonstrate that participants who watched the no-narration video and the pro-persecution video were more likely to think that the establishment bodies functioned more properly after watching the videos. Participants who watched the pro-defense video were more likely to think that the functioning of the establishment bodies was more problematic. (The findings regarding the police were significant for all videos, the findings for the prosecution and the court were significant in some videos, but the direction of change was the same).

## 6 Discussion and conclusions

The proliferation of user-generated videos has reshaped the landscape of information dissemination, particularly within the realm of activism and anti-establishment movements.

In particular, people's enhanced abilities to produce videos is a part of the broader Do-It-Yourself (DIY) trend. This trend is useful for oppositional and anti-establishment groups, paving the way for “video exchanges” between citizens and establishment. Does watching such videos indeed change minds? Can it produce significant changes in-line with narratives promoted by activists or by the establishment?

This study delved into the effects of such videos on public opinion, shedding light on their capacity to influence views both directly and indirectly. The paper focuses on videos due to their enhanced impacts on viewers, and importance in the legal and activism domains. In the experiment we analyzed viewers' opinion-change about contents that *appears* and *does not appear* in the videos. We found that:

• People who watched the no-narration and pro-prosecution videos were more likely to think Zadorov was guilty after watching the video. But participants who watched the pro-defense video were more likely to support his innocence,
• People who watched the no-narration and pro-prosecution videos were more likely to think that the establishment bodies functioned properly after watching the video. But participants who watched the pro-defense video were more likely to think that the functioning of the establishment bodies was problematic.

Viewers' opinions changed regarding Zadorov's innocence/guilt and the functioning of the police, which were explicitly addressed in the videos. Interestingly, the effect carried-over to the functioning of the state attorney's office and the courts-although the videos contained no reference to them. The findings illustrate the power of videos: watching them affects not only opinion about issues they address, but it also carries over to opinions about issues they do not address.

As we discuss the implications and limitations of our findings, we draw upon existing scientific literature to contextualize our results and provide a more comprehensive understanding of the dynamics at play.

Our findings align with previous studies that have explored the persuasive power of visual content in shaping attitudes ([7,8]; Paivio & Csapo, 1993). Furthermore, our study has revealed a noteworthy carryover effect, extending beyond the boundaries of video content to influence perceptions of state institutions, such as the police, state attorney's office, and the courts. This phenomenon corroborates prior research emphasizing the potential of visual content to induce opinion change in related domains [11,15,29].

The implications of these findings are far-reaching, especially in the contemporary media landscape where individuals and grassroots organizations wield the ability to produce and distribute videos with ease. The capacity to sway public opinion through visual materials has profound implications for activism, as well as the broader dissemination of information. Our research suggests that exposure to such materials may significantly impact viewers, reshaping their perspectives on issues far beyond the scope of the presented content.

Nonetheless, it is essential to acknowledge the limitations of our study, including our sample size and the potential for biases within our experimental design. Future research should seek to address these limitations, while also delving deeper into the mechanisms that underlie the carryover effects observed in our study. Additionally, further investigation is warranted to explore how institutional bodies and private individuals navigate the creation and interpretation of videos in their efforts to influence public opinion. For example, it seems that the ability of institutional bodies to add interpretive layers to videos is limited, since such actions can be perceived as a “blatant attempt to skew public opinion"- while private people can arguably “do as they please”. These developments contribute to strengthening trends of decentralization, pluralization, and the influence of niche and anti-establishment organizations and groups on public opinion.

In conclusion, our study illuminates the transformative power of videos in contemporary information ecosystems, emphasizing their ability to shape opinions and potentially challenge established narratives. This research contributes to the evolving field of media effects, underlining the significance of visual content in a world where decentralized, grassroots activism holds increasing sway over public opinion.

## Data availability statement

The data upon which this study is based can be provided upon request.

## CRediT authorship contribution statement

**Azi Lev-On:** Writing – review & editing, Writing – original draft, Visualization, Validation, Supervision, Resources, Project administration, Methodology, Investigation, Funding acquisition, Formal analysis, Data curation, Conceptualization. **Nili Steinfeld:** Writing – original draft, Visualization, Validation, Supervision, Software, Resources, Methodology, Investigation, Funding acquisition, Formal analysis, Data curation, Conceptualization.

## Declaration of competing interest

The authors declare that they have no known competing financial interests or personal relationships that could have appeared to influence the work reported in this paper.
