# Supplementary material for: Carry-over effects of priming viewers with pro and anti-establishment messages in video content
Source: Heliyon. 2024 Mar 15;10(7):e27895. doi: 10.1016/j.heliyon.2024.e27895 (PMC10981022; doi:10.1016/j.heliyon.2024.e27895)
Supplement: Multimedia component 1 [file mmc1.docx]

**Questionnaire before (translated from Hebrew)**

Tair Rada, a young girl, was murdered in 2006. Roman Zadorov was found guilty of her murder in 2010, and his appeal was rejected in 2015. The public has continued discussing matters relating to the murder and the trial Until today.

How much do you feel you know about the case? From 1 to 7, please select:

| 7- Highly knowledgeable | 6 | 5 | 4 | 3 | 2 | don't know at all- 1 |
| --- | --- | --- | --- | --- | --- | --- |

(Buffer Question) A wounded terrorist was killed on March 24, 2016, when Sergeant Alor Azaria shot him in the head. He was found guilty of manslaughter and inappropriate behavior in an early 2017 military court. His acts, Azaria's indictment and sentencing, and the IDF's and other political figures' responses, all sparked intense public debate in Israel.

How much do you feel you know about the case? From 1 to 7, please select:

| 7- Highly knowledgeable | 6 | 5 | 4 | 3 | 2 | don't know at all- 1 |
| --- | --- | --- | --- | --- | --- | --- |

1. For each of the following questions on the Roman Zdorov trial, select an answer ranging from 1 (not at all) to 7 (very much). Answer 0 if it's not relevant.

|  |  | not at all |  |  |  |  |  | very much | irelevant |
| --- | --- | --- | --- | --- | --- | --- | --- | --- | --- |
| 1 | How much does this affair interest you? | 1 | 2 | 3 | 4 | 5 | 6 | 7 | 0 |
| 2 | To what extent have you been exposed to information from major media sources (press, radio, television, and news websites (such as YNET, WALLA, NRG, etc.) on the subject? | 1 | 2 | 3 | 4 | 5 | 6 | 7 | 0 |
| 3 | To what extent do you disclose information from Facebook groups that are dealing with this? | 1 | 2 | 3 | 4 | 5 | 6 | 7 | 0 |
| 4 | How much do you think about the subject? | 1 | 2 | 3 | 4 | 5 | 6 | 7 | 0 |
| 5 | How much have you talked about this subject with your friends/family? | 1 | 2 | 3 | 4 | 5 | 6 | 7 | 0 |
| 6 | How much have you tried to convince others about your opinion? | 1 | 2 | 3 | 4 | 5 | 6 | 7 | 0 |

2. For every claim below, please indicate your position on a scale from 1 (greatly disagree) to 7 (greatly agree). If you don’t have a position on the subject, reply 0.

|  |  | greatly disagree |  |  | I disagree and agree equally |  |  | greatly agree | I have no position |
| --- | --- | --- | --- | --- | --- | --- | --- | --- | --- |
| 1 | Roman Zedorov murdered Tair Radda. | 1 | 2 | 3 | 4 | 5 | 6 | 7 | 0 |

3. Regarding the trial of Roman Zedorov - for the claims below, please indicate your position on a scale from 1 (worst) to 7 (best). If you don’t have a position on the subject, reply 0:

|  |  | The worst |  |  |  |  |  | The best | I have no position |
| --- | --- | --- | --- | --- | --- | --- | --- | --- | --- |
| 4 | Based on your knowledge, what is your position on the conduct **of the** **police** in this case (1- worst, 7-best) |  |  |  |  |  |  |  |  |
| 5 | Based on your knowledge, what is your position on the conduct **of the** **prosecutor**s in this case (1 worst, 7 best) |  |  |  |  |  |  |  |  |
| 6 | Based on your knowledge, what is your position on the conduct **of the court** in this case (1- worst, 7-best) |  |  |  |  |  |  |  |  |
