# Supplementary material for: Carry-over effects of priming viewers with pro and anti-establishment messages in video content
Source: Heliyon. 2024 Mar 15;10(7):e27895. doi: 10.1016/j.heliyon.2024.e27895 (PMC10981022; doi:10.1016/j.heliyon.2024.e27895)
Supplement: Multimedia component 2 [file mmc2.docx]

**Questionnaire AFTER (translated from Hebrew)**

For every claim below, please indicate your position on a scale from 1 (greatly disagree) to 7 (greatly agree). If you don’t have a position on the subject, reply 0.

|  |  | greatly disagree |  |  | I disagree and agree equally |  |  | greatly agree | I have no position |
| --- | --- | --- | --- | --- | --- | --- | --- | --- | --- |
| 1 | Roman Zedorov murdered Tair Radda. | 1 | 2 | 3 | 4 | 5 | 6 | 7 | 0 |

Regarding the trial of Roman Zedorov - for the claims below, please indicate your position on a scale from 1 (worst) to 7 (best). If you don’t have a position on the subject, reply 0:

|  |  | The worst |  |  |  |  |  | The best | I have no position |
| --- | --- | --- | --- | --- | --- | --- | --- | --- | --- |
|  | Based on your knowledge, what is your position on the conduct **of the** **police** in this case (1- worst, 7-best) |  |  |  |  |  |  |  |  |
|  | Based on your knowledge, what is your position on the conduct **of the** **prosecutor**s in this case (1 worst, 7 best) |  |  |  |  |  |  |  |  |
|  | Based on your knowledge, what is your position on the conduct **of the court** in this case (1- worst, 7-best) |  |  |  |  |  |  |  |  |

3. A few minutes ago, you saw a video composed of parts of the reconstructions of the murder. Is it for you (yes or no):

a. This is the first time you have been exposed to this video, including the murder film.

b. previously been exposed to videos of the murder re-enactment, but not to this part.

c. previously exposed to this section of the murder reconstruction - but with a different editing.

d. previously exposed to this section of the murder reconstruction - in a similar or identical edit

4. Please answer about the video you watched (between 1 and 7) for your opinion:

| The video is professionally produced | 1 | 2 | 3 | 4 | 5 | 6 | 7 | The video was produced amateurishly |
| --- | --- | --- | --- | --- | --- | --- | --- | --- |
| The video was reliably produced | 1 | 2 | 3 | 4 | 5 | 6 | 7 | The video was not reliably produced |
| The content of the video is unclear | 1 | 2 | 3 | 4 | 5 | 6 | 7 | The content of the video is clear |
| The video is convincing | 1 | 2 | 3 | 4 | 5 | 6 | 7 | The video is unconvincing |
| The video is boring | 1 | 2 | 3 | 4 | 5 | 6 | 7 | The video is interesting |
| The video content is unreliable | 1 | 2 | 3 | 4 | 5 | 6 | 7 | The video content is reliable |
| The video inspires thought | 1 | 2 | 3 | 4 | 5 | 6 | 7 | The content of the video is not mind-provoking |
| The video makes me want to explore deeper | 1 | 2 | 3 | 4 | 5 | 6 | 7 | The video doesn't make me want to explore deeper |

5. How much viewing the video has disclosed new information for you:

|  | 1- Very renewed | 2 | 3 | 4 | 5 | 6 | 7- Not renewed at all |
| --- | --- | --- | --- | --- | --- | --- | --- |
| About the murder case |  |  |  |  |  |  |  |
| Police conduct in the case |  |  |  |  |  |  |  |
| About the behavior of Roman Zadarov in the case |  |  |  |  |  |  |  |

6. Do you think you might be interested in the future:

|  | 1- Not interested | 2 | 3 | 4 | 5 | 6 | 7- Very interested |
| --- | --- | --- | --- | --- | --- | --- | --- |
| watch the video presented to you again |  |  |  |  |  |  |  |
| See more videos about the case |  |  |  |  |  |  |  |
| Read more journalistic materials (articles and investigations) about the case |  |  |  |  |  |  |  |
| Read legal materials on the subject - court rulings, High Courts regarding the case |  |  |  |  |  |  |  |
| Read additional materials, such as court records and testimony regarding the case |  |  |  |  |  |  |  |
